# Pituitary Adenoma Masquerading as Diabetic Third Nerve Palsy

**DOI:** 10.7759/cureus.60037

**Published:** 2024-05-10

**Authors:** Renu Magdum, Kalpita B Goli, Aparna Alapati, Aditya Ganesh, Sindhu Kilari

**Affiliations:** 1 Ophthalmology, Dr. D. Y. Patil Medical College, Hospital and Research Centre, Pune, IND

**Keywords:** bitemporal hemianopia, diabetes, oculomotor nerve palsy, pituitary macroadenoma, cranial nerve palsies

## Abstract

A 46-year-old uncontrolled diabetic female visited the ophthalmology outpatient department with a sudden onset of drooping of the upper lid and restriction of movements in adduction, depression, and elevation in the right eye, suggestive of third nerve palsy. Initially, it was thought to be due to a vasculogenic cause due to uncontrolled diabetes, but visual fields revealed bitemporal hemianopia, characteristic of a pituitary adenoma. The diagnosis was confirmed by a CT scan. The patient then underwent a trans-nasal endoscopic removal of the pituitary macroadenoma, followed by a partial recovery of vision.

## Introduction

Oculomotor nerve palsies can develop from various causes, such as vascular disease, trauma, aneurysms, or intracranial tumors. Neuropathies affecting cranial nerves are common complications of unmanaged type 2 diabetes mellitus, with prevalence linked to the extent and duration of high blood sugar levels. Studies indicate approximately 0.75-1% of diabetic patients develop cranial neuropathy. Within this group, about 11% experience oculomotor nerve palsy as a consequence of diabetes. Neuropathies are well-known complications of uncontrolled type 2 diabetes mellitus, and the prevalence depends on the severity as well as the duration of hyperglycemia. Different studies have shown that about 0.75-1% of patients with diabetes mellitus eventually develop cranial neuropathy [1]. Among the patients with mononeuropathies, it has been reported that oculomotor nerve palsy secondary to diabetes occurs in 11% of patients [2]. Pituitary adenomas constitute 12-15% of symptomatic intracranial neoplasms. Bitemporal hemianopia has been described as a hallmark sign [3,4]. This is due to tumor extension into the adjacent cavernous sinus due to sellar compression. The occurrence of ocular motor nerve palsies in pituitary tumors ranges from 14% to 32%, predominantly affecting the third nerve (seven out of nine cases), often accompanied by additional cranial nerve involvement [5].

## Case presentation

A 46-year-old woman with uncontrolled diabetes for eight years presented to the ophthalmology clinic with sudden-onset drooping of the right upper lid associated with headaches for 15 days. Past medical history: she had a known case of diabetes mellitus for 8 years and was on the following medications: tablet Metformin (400 mg) + Glibenclamide (2.5 mg) twice a day, tablet Glimepiride (1 mg) twice a day, and an injection of Human Mixtard (13 units) at night. However, she has a history of not taking the medications regularly.

**Figure 1:**
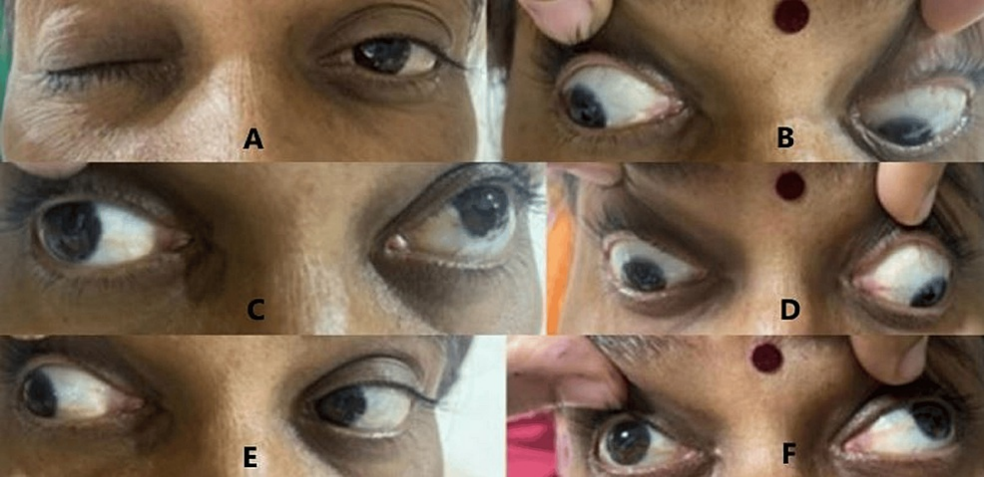
On presentation, right ptosis with restriction of all movements except lateral rectus, suggestive of complete third nerve palsy (A) Right eye complete ptosis, (B) right eye restriction in down gaze, (C) right eye restriction in elevation, (D) right eye restriction in down and outward gaze, (E) right eye normal lateral gaze, (F) right eye restriction in up and inward gaze

Ocular examination showed restriction in right eye adduction, depression, and elevation movements, as shown in Figure 1B-1F, with complete ptosis, as shown in Figure 1A. The pupils were unaffected. No other cranial nerve was affected. The fundus examination was normal in both eyes. These findings were suggestive of a pupil-sparing third nerve palsy in the right eye, probably due to uncontrolled diabetes.

Blood sugar tests confirmed uncontrolled diabetes mellitus (fasting and postprandial plasma glucose were 396 mg/dL and 440 mg/dL, respectively), with an HbA1C of 11.2% (diabetic > 6.4%).

Visual acuity was normal in both eyes. Both eyes were 6/36 to >6/6 (−1.00DS), and near vision was N12 to >N6 (with an add of +1.50DS). This was surprising, as on elevating the affected upper lid, the patient did not complain of any diplopia. This prompted a visual field examination. Visual field testing done using a Humphrey Automated Perimeter revealed bitemporal hemianopia, suggestive of chiasmal compression.

**Figure 2:**
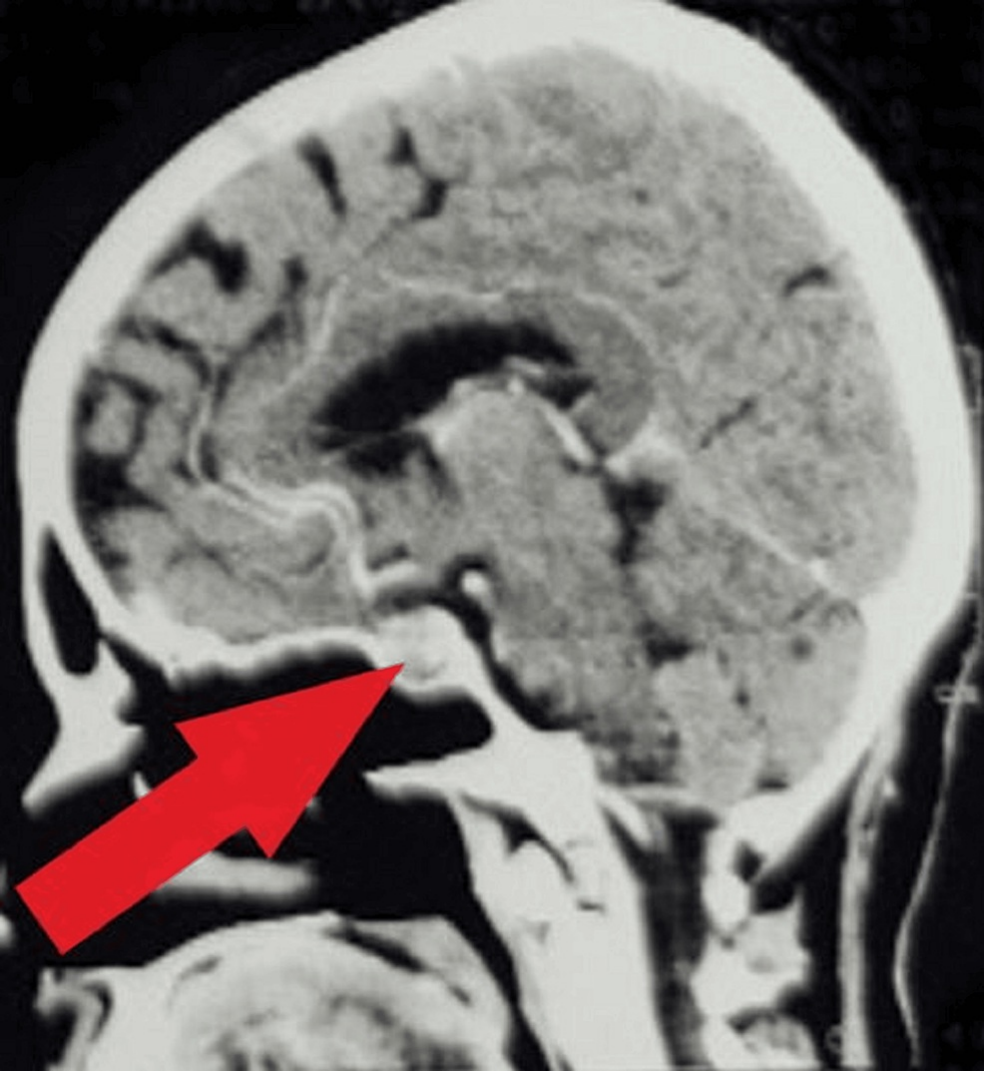
A well-defined isodense to hyperdense lesion was noted in the suprasellar and sellarcistern causing widening of sellar turcica and seen indenting the 3rd, 4th, and 5th nerves on right side The red arrow indicated pitutary macrodenoma

CT brain scan results uncovered a distinct, well-defined, heterogeneously enhancing hypodense mass lesion in the sellar region, indicating a pituitary macroadenoma, as shown in Figure 2. Additionally, an isodense to hyperdense lesion was identified in the suprasellar and sellar cistern, causing widening of the sella turcica and impacting the third, fourth, and fifth nerves on the right side.

Despite MRI being more sensitive, CT was chosen due to affordability concerns for the patient. The patient was diagnosed with a pituitary macroadenoma and underwent trans-nasal endoscopic pituitary adenoma removal.

**Figure 3:**
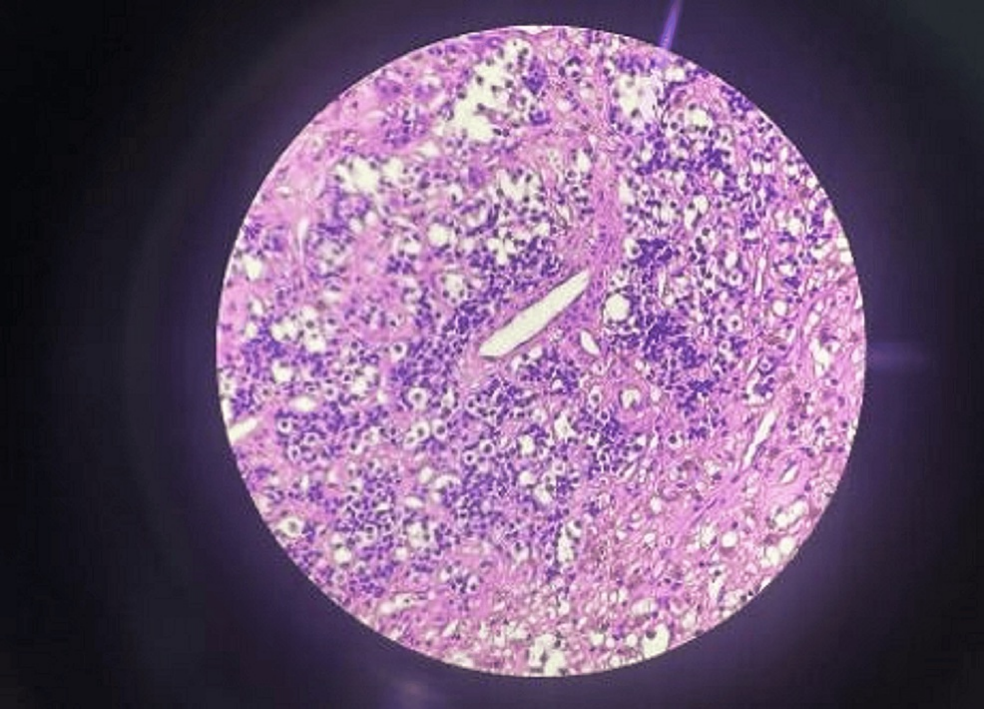
HPE showed monomorphic cells with lack of reticular network among neoplastic cells arranged in nest and sheets pattern suggestive of pituitary macroadenoma The stain used is hematoxylin and eosin, and resolution power used under the microscope was 40×

The lesion exhibited a reddish-pink hue and a soft consistency, readily amenable to suction. A complete excision of the lesion was performed, resulting in the removal of the entire mass. The mass was sent for histopathological examination, as shown in Figure 3, which was suggestive of pitutary macroadenoma.

**Figure 4:**
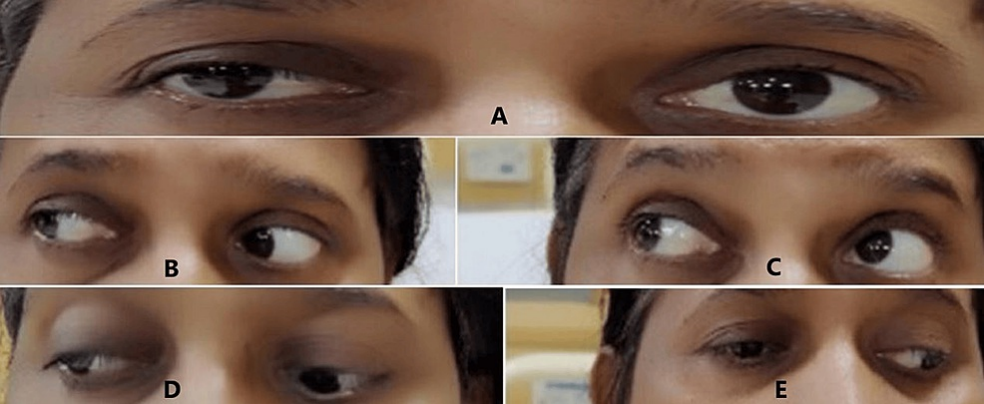
Post-op photo showing partial resolution of ptosis and good recovery of ocular movements (A) Improvement in the ptosis in the right eye; (B) right-eye normal lateral gaze; (C) right-eye normal upward gaze; (D) right-eye normal down and outward gaze; (E) right-eye normal down and inward gaze

Following surgery, the patient experienced a smooth recovery with complete resolution of ptosis, as shown in Figure 4A-4E, without any occurrence of diabetes insipidus or postoperative leaks.

## Discussion

The oculomotor nerve is commonly involved in diabetic mononeuropathies. Microvascular ischemia is the most common cause of oculomotor and abducens palsy, with 58.1% and 69.8%, respectively [6,7]. Pituitary adenomas comprise about 10% of intracranial tumors [8]. Around 70% of pituitary adenomas exhibit visual symptoms, often characterized by reduced visual acuity and/or field defects. Other presentations include headaches and hormonal imbalances. As pituitary adenomas enlarge, they have the potential to compress adjacent structures such as the optic chiasma and cranial nerves in the cavernous sinus. In a retrospective study of 29 cases involving pituitary tumors with suprasellar extension, it was revealed that bitemporal hemianopia was the predominant observation [9]. In a review of 12 patients with pituitary adenomas with accompanying cranial nerve palsies, the third cranial nerve exhibited the highest frequency of involvement, followed by the sixth and fourth cranial nerves. The symptom sequence of third cranial nerve palsy typically begins with mydriasis, followed by limited eye movement and ptosis. Recovery typically occurs in the reverse order of symptom development. An early surgical intervention was advocated by the authors [10]. Lau et al. documented a pituitary adenoma case marked by complete bilateral oculomotor nerve palsies, accompanied by minimal visual ﬁeld impairment, with the abducens and trochlear nerve remaining intact. Despite surgical tumor removal, visual acuity in both eyes remained at 6/18 [9].

A thorough understanding of the differential diagnosis is essential for accurate diagnosis and appropriate management. Compression of the oculomotor nerve can occur due to tumors or intracranial masses that may exert pressure on the nerve, leading to dysfunction. Direct trauma to the head or orbit can result in third nerve palsy. Inflammatory disorders such as Tolosa - Hunt syndrome or sarcoidosis may involve the oculomotor nerve, leading to palsy. Infections affecting the central nervous system, such as meningitis, or viral infections like herpes zoster, can manifest as third nerve palsy. Vasculopathic conditions, including atherosclerosis or vasculitis, may lead to impaired blood flow to the oculomotor nerve, resulting in palsy.

We highlight an atypical presentation of pituitary macro-adenoma masquerading as diabetic oculomotor nerve mononeuropathy. The occurrence of isolated third nerve palsy is a rare initial presentation of pituitary adenoma. Typically, limitations in movement are often attributed to unmanaged diabetes in such cases. However, a thorough examination reveals that the eye impairment stems directly from the tumor itself. The fundus is usually normal, as the pathology is at the chiasm level and not at the level of the optic disc. The absence of diplopia in spite of normal visual acuity in both eyes prompted a visual field examination that revealed bitemporal hemianopia and took us in the right direction. In subsequent follow-ups, the patient's ptosis resolved, and there was partial alleviation of the previously observed restriction of extraocular movements. The patient was advised regarding proper control of blood sugar levels and regular follow-up.

## Conclusions

In diabetic patients presenting with third nerve palsy, timely assessment and intervention are paramount to mitigate potential complications and optimize outcomes. This neurological deficit necessitates a comprehensive evaluation to discern between diabetic microvascular changes and other potential vascular or compressive etiologies. Management strategies should address both the acute neurological manifestations and the underlying diabetic pathology. Therefore, a multidisciplinary approach involving neurologists, ophthalmologists, and endocrinologists is often essential to ensure thorough care. Understanding the intricate association between diabetes and third nerve palsy underscores the importance of proactive diabetic management to prevent and effectively manage neurological complications. By addressing both the acute neurological deficit and the systemic diabetic pathology, clinicians can strive to improve the quality of life for diabetic patients affected by third nerve palsy.
